# Comparative evaluation of total mandibular inferior border circumferential osteotomy and T-chinoplasty with mandibular angle osteotomy

**DOI:** 10.1590/1806-9282.20251933

**Published:** 2026-06-29

**Authors:** Junwen Xiong, Wenjie Xu, Min Wei, Dongkao He

**Affiliations:** 1 Shanghai Ren’ai Hospital, Department of Plastic Surgery and Beauty – Shanghai, China.; 2 Shanghai Xi’ai Medical, Beauty Clinic – Shanghai, China.; 3 Shanghai Ninth People’s Hospital, Department of Plastic Surgery – Shanghai, China.

**Keywords:** Orthognathic surgery, Osteotomy, Mandible, Genioplasty, Maxillofacial abnormalities

## Abstract

**OBJECTIVE:**

The aim of this study was to compare clinical outcomes, patient satisfaction, and postoperative complications between total mandibular inferior border circumferential osteotomy and T-shaped chin contouring combined with mandibular angle osteotomy, and to evaluate their surgical indications.

**METHODS:**

A retrospective analysis was conducted on 51 patients who underwent mandibular contouring between January 2018 and January 2022. According to the surgical technique, 21 patients were assigned to total mandibular inferior border circumferential osteotomy (Group I) and 30 to T-shaped chin contouring with mandibular angle osteotomy (Group II). Pre- and postoperative computed tomography images were analyzed to assess changes in chin width, height, offset, and facial proportions. Patient satisfaction and postoperative complications were also evaluated.

**RESULTS:**

Postoperative complications included lower lip numbness, hematoma, infection, and soft tissue ptosis, with no significant difference between the two groups (all p>0.05). Satisfaction rates were similarly high in both groups (mean scores: 4.58±0.42 vs. 4.72±0.56, p>0.05). However, Group I achieved a significantly greater chin width reduction and narrowing rate than Group II (p<0.01), with a smaller postoperative chin offset (p<0.01). Group I patients also showed improved facial symmetry and proportion, with no significant difference in postoperative chin-to-midface height ratio (p>0.05).

**CONCLUSION:**

Both techniques provide favorable aesthetic and functional results. Total mandibular inferior border circumferential osteotomy yields greater chin narrowing and better midline alignment, making it particularly suitable for patients with long, wide, or asymmetric lower faces seeking improved mandibular contour.

## INTRODUCTION

Total mandibular inferior border circumferential osteotomy and T-chinoplasty are widely utilized surgical techniques in oral and maxillofacial surgery to correct jaw deformities and enhance facial aesthetics. In contrast, mandibular angle osteotomy is a specific procedure designed to modify the mandibular angle and contour of the lower jaw. Baek et al.^1^ first reported the application of mandibular angle osteotomy for mandibular contouring. Since then, various modifications—such as curved mandibular angle osteotomy and “V-line” osteotomy—have been developed to improve the correction of square jaw deformities^2^.

However, the chin constitutes a critical aesthetic component of the lower third of the face. In patients with square jaws and broad chins, a simple mandibular angle osteotomy or even a “V-line” osteotomy often fails to achieve sufficient chin narrowing, as the osteotomy range terminates at the mandibular body or chin region. Consequently, postoperative outcomes may remain unsatisfactory. To address this clinical limitation, both T-chinoplasty and total mandibular inferior border circumferential osteotomy were introduced. T-chinoplasty, a classical and widely adopted technique, particularly in Korea^3^, involves one horizontal and two vertical osteotomy lines in the chin, allowing for bone grafting to lengthen the vertical height, advance or retract the chin sagittally, and adjust its horizontal width.

Total mandibular inferior border circumferential osteotomy corrects mandibular asymmetry and disproportion by resecting portions of the mandibular border and repositioning the remaining bone segments with circumferential fixation. Conversely, T-chinoplasty improves zygomatic-chin overhang and mandibular contour irregularities by resecting part of the zygomatic arch and remodeling the mandible through reconstruction and fixation^4^.

Despite the clinical utility of these procedures, direct comparative analyses between total mandibular inferior border circumferential osteotomy and T-shaped chinoplasty combined with mandibular angle osteotomy remain limited. Therefore, this study aimed to compare these two surgical approaches by evaluating their clinical outcomes, postoperative facial appearance, functional recovery, and patient satisfaction to determine their respective advantages and indications in mandibular deformity correction. However, direct comparative analyses between these two approaches remain scarce. Given their distinct anatomical scopes, surgical complexity, and indications—total osteotomy for broad/asymmetric chins versus T-chinoplasty for short/retruded chins with angle prominence—a systematic comparison is clinically warranted to guide individualized surgical planning and optimize outcomes.

## METHODS

### Study population

A retrospective analysis was performed on patients who underwent mandibular contouring surgery at the Department of Plastic Surgery of our affiliated hospitals between January 2018 and January 2022. According to the surgical method, patients were assigned to two groups: total mandibular inferior border circumferential osteotomy (Group I, n=21) and T-shaped chin contouring combined with mandibular angle osteotomy (Group II, n=30).

Inclusion criteria were as follows: (1) patients who underwent either of the two surgical procedures; (2) complete clinical and imaging data with a minimum postoperative follow-up of 6 months; and (3) age ≥18 years without other maxillofacial abnormalities. Exclusion criteria included: (1) systemic diseases; (2) maxillofacial tumors or fractures; (3) congenital craniofacial malformation syndromes; and (4) prior mandibular contouring surgery.

### Data collection

Preoperative and final postoperative craniofacial computed tomography (CT) images were collected for each patient. Postoperative outcomes were evaluated through the analysis of facial height, chin width, chin offset, facial proportions, patient satisfaction, and complications. Follow-up was conducted for 6–24 months post-surgery.

Patient satisfaction was assessed using a five-point questionnaire: very satisfied (5 points), satisfied (4), average (3), dissatisfied (2), and very dissatisfied (1). Scores were averaged to obtain the mean satisfaction value for each group.

### Postoperative complications

All postoperative complications were recorded and compared between groups. These included lower lip numbness, hematoma, severe swelling, postoperative infection, and soft tissue ptosis. The incidence of each complication was expressed as a percentage of the total number of patients per group.

### Evaluation of surgical outcomes

Objective evaluation indices included pre- and postoperative measurements of chin width, chin offset, chin height, and facial proportions. The facial proportion parameters consisted of the midface height-to-chin height ratio and the chin width-to-chin height ratio.

Three-dimensional craniofacial reconstructions were created using *Mimics 21.0* (Materialise, Belgium). Superimposed pre- and postoperative CT images were analyzed to quantify changes in mandibular shape. The Frankfort Horizontal Plane (FHP) served as the horizontal reference line. For chin width measurement, vertical lines were drawn from the proximal roots of the first mandibular molars on both sides to intersect the anterior-inferior border of the mandible, defining points A (right) and B (left). Postoperatively, the intersections of the same reference line with the altered mandibular margin were marked A′ and B′. The distances AB and A′B′ represented pre- and postoperative chin widths, respectively.

The mid-sagittal plane (MSP), passing through the nasion (N), sella (S), and anterior nasal spine (ANS), was used as the vertical reference plane. Chin deviation (offset) was measured as the perpendicular distance between the chin point (Menton, Me) and the MSP before and after surgery (Me and Me′). Additionally, *3-matic 12.0* (Materialise, Belgium) software was used to overlap mirror images of the mandible to visualize and assess asymmetry correction qualitatively.

Lower face height was defined as the vertical distance between ANS and Me, while midface height was measured between N and ANS. The chin width reduction ratio was calculated as:


$$\text{Chin width reduction ratio}=\frac{\left(AB-A\prime B\prime \right)}{AB}\times 100\%$$

All measurements were performed by two independent observers, and mean values were used for analysis.

### Definition of terminology and measurement landmarks

For clarity and consistency, the following terms are defined: Chin deviation (offset): The perpendicular distance from the Menton (Me) to the MSP. Narrowing ratio: Calculated as: $\text{Chin width reduction ratio}=\frac{\left(AB-A\prime B\prime \right)}{AB}\times 100\%$ Lower facial height: The vertical distance from ANS to Me. Midfacial height: The vertical distance from N to ANS. All measurements were referenced to the FHP and MSP.

### Statistical analysis

Data were analyzed using the Statistical Package for the Social Sciences *20.0* (IBM Corp., Armonk, NY, USA). Continuous variables conforming to a normal distribution were expressed as mean±standard deviation (x̄±*s*), while categorical data were presented as counts and percentages.

Comparisons between the two groups for continuous variables—including age, satisfaction scores, chin width (AB, A′B′), chin offset (Me–MSP, Me′–MSP), chin height (ANS–Me, ANS–Me′), midface height (N–ANS), and various facial ratios—were performed using the independent-samples t-test. Categorical variables such as sex distribution and postoperative complications were analyzed using the chi-square test. A p<0.05 was considered statistically significant.

## RESULTS

### Comparison of general information

The general clinical characteristics of the two groups were comparable. Group I (total mandibular inferior border circumferential osteotomy) included 21 patients (12 males and 9 females; mean age, 29.27±6.45 years), while Group II (T-shaped chin contouring combined with mandibular angle osteotomy) comprised 30 patients (12 males and 18 females; mean age, 28.13±5.84 years). No statistically significant differences were observed between the two groups regarding age or sex distribution (p>0.05). The mean postoperative follow-up period was 8.38±2.64 months. All patients achieved satisfactory healing without major complications such as mandibular fracture or bone displacement.

### Comparison of postoperative complications

Postoperative complications in Group I included two cases of lower lip numbness (9.52%), one case of hematoma (4.76%), two cases of postoperative infection (9.52%), and one case of soft tissue ptosis (4.76%).

In Group II, complications included three cases of lower lip numbness (10.00%), two cases of hematoma (6.67%), two cases of postoperative infection (6.67%), and two cases of soft tissue ptosis (6.67%).

There were no statistically significant differences in the overall incidence or distribution of complications between the two groups (p>0.05; Table 1). All complications were managed conservatively, and no patient required reoperation.

**Table 1:** Comparison of postoperative complications and postoperative satisfaction between two groups [n(%)].

Group	Postoperative complications				Postoperative satisfaction			
	Lower lip numbness	Hematoma	Postoperative infection	Soft tissue ptosis	Very satisfied [n(%)]	Satisfied [n(%)]	Fair [n(%)]	Mean satisfaction rating (x̄s)
I group (n = 21)	2 (9.52)	1 (4.76)	2 (9.52)	1 (4.76)	16 (76.19)	3 (14.29)	2 (9.52)	4.58±0.42
II group (n = 30)	3 (10.00)	2 (6.67)	2 (6.67)	2 (6.67)	22 (73.33)	7 (23.33)	1 (3.33)	4.72±0.56
χ^2^ value	0.24	0.68	0.17	1.05	0.57	0.78	0.84	0.926
p-value	0.102	0.471	0.302	0.106	0.568	0.603	0.205	0.628

### Comparison of postoperative satisfaction

Postoperative satisfaction was high in both groups. In Group I, 16 patients (76.19%) were very satisfied, 3 (14.29%) were satisfied, and 2 (9.52%) rated their outcomes as average. In Group II, 22 patients (73.33%) were very satisfied, 7 (23.33%) were satisfied, and 1 (3.33%) reported an average outcome.

The mean satisfaction score was 4.58±0.42 in Group I and 4.72±0.56 in Group II, with no statistically significant difference between the groups (p>0.05; Table 1).

### Comparison of chin width and chin offset before and after surgery

Postoperative analysis demonstrated that Group I achieved significantly greater chin width reduction and narrowing rate compared with Group II (p<0.01).

Preoperative chin offset did not differ significantly between the two groups (p>0.05). However, postoperative chin offset was markedly smaller in Group I than in Group II (p<0.01), indicating improved midline alignment in patients who underwent total mandibular inferior border circumferential osteotomy.

Superimposed CT imaging confirmed that postoperative chin deviation was reduced to a greater extent in Group I compared with Group II (Table 2).

**Table 2:** Comparison of chin width and chin offset before and after surgery [(x±s)].

Measurement index	I group (n=21)	II group (n=30)	t-value	p-value
Chin width				
Preoperation (mm)	56.29±2.79	55.78±3.03	2.18	0.002
Postoperation (mm)	43.78±3.52	46.09±3.25	-2.75	0.013
Narrowing amount (mm)	12.51±2.68	9.69±2.86	7.26	0.001
Narrowing rate (%)	21.14±3.78	15.26±3.05	6.09	0.002
Chin offset				
Preoperation (mm)	0.52±0.26	0.58±0.24	-1.58	0.178
Postoperation (mm)	0.16±0.05	0.35±0.21	-2.57	0.023

Notes: Point A and B: the right and left preoperative intersection points of a line perpendicular to the Frankfort horizontal plane through the mesial apex of the mandibular first molar and the anterior inferior border of the mandible. Point A′ and B′: the right and left postoperative intersection points of line AB and the anterior inferior border of the mandible. AB-A′B: the amount of chin narrowing. [(AB-A′B)/AB]. The ratio of chin narrowing. Me, menton point. ANS, anterior nasal spine N; nasion point; amount of chin deviation. Me-MSP: lower facial height. N-ANS: midfacial height. Surgery does not affect midfacial height. Only preoperative midfacial height is measured.

### Comparison of facial computed tomography measurements before and after surgery

Preoperative facial CT measurements revealed that patients in Group I exhibited significantly greater chin height and a higher chin-to-midface height ratio compared with those in Group II (p<0.01). However, postoperative chin-to-midface height ratios did not differ significantly between groups (p>0.05), suggesting comparable vertical facial balance after surgery.

Both preoperative and postoperative chin width-to-lower face height ratios were significantly smaller in Group I than in Group II (p<0.01), indicating a more favorable narrowing effect and harmonious lower facial contour achieved by total mandibular inferior border circumferential osteotomy (Table 3).

**Table 3:** Comparison of facial computed tomography values before and after surgery.

Measurement index	I group (n=21)	II group (n=30)	t-value	p-value
Lower facial height				
Preoperation (mm)	62.89±4.26	53.08±4.27	8.96	0.012
Postoperation (mm)	57.85±4.18	57.37±3.89	0.73	0.527
Midfacial facial height^*^: (preoperative, mm)				
Lower facial height: midfacial height (%)				
Preoperation (mm)	107.47±3.28	91.58±7.69	10.57	0.001
Postoperation (mm)	98.82±1.84	99.27±1.69	1.56	0.079
Chin width: lower facial height (%)				
Preoperation (mm)	90.26±3.57	103.62±5.35	-12.78	0.006
Postoperation (mm)	76.56±1.97	80.57±3.28	-5.78	0.003

Notes: Point A and B: the right and left preoperative intersection points of a line perpendicular to the Frankfort horizontal plane through the mesial apex of the mandibular first molar and the anterior inferior border of the mandible. Point A′ and B′: the right and left postoperative intersection points of line AB and the anterior inferior border of the mandible. AB-A′B: the amount of chin narrowing. [(AB-A′B)/AB]. The ratio of chin narrowing. Me, menton point. ANS, anterior nasal spine. N, nasion point; amount of chin deviation. Me-MSP: lower facial height. N-ANS: midfacial height. *Surgery does not affect midfacial height. Only preoperative midfacial height is measured.

In clinical practice, a chin deviation>2 mm is generally considered visually noticeable and clinically relevant. In our cohort, postoperative chin deviation in both groups was well below this threshold, indicating both techniques effectively correct asymmetry to a clinically acceptable level.

## DISCUSSION

In this study, we compared the clinical outcomes of total mandibular inferior border circumferential osteotomy and T-chinoplasty combined with mandibular angle osteotomy for the correction of mandibular deformities. Our findings demonstrated that both surgical techniques achieved satisfactory aesthetic and functional outcomes. Each procedure effectively corrected mandibular asymmetry, reduced chin ptosis, and improved the overall mandibular contour^5^. These results indicate that both methods are reliable options for mandibular contouring. However, the optimal surgical choice should be determined by considering each patient’s anatomical features and aesthetic goals^6^.

Total mandibular inferior border circumferential osteotomy was found to achieve a greater reduction in chin width and a higher narrowing efficiency compared with T-chinoplasty combined with mandibular angle osteotomy (p<0.001). The postoperative chin offset was also smaller, indicating superior midline symmetry. These results suggest that total mandibular inferior border circumferential osteotomy is particularly effective for patients with a long or broad chin, as it simultaneously reduces lower face height and narrows the chin. In contrast, T-chinoplasty combined with mandibular angle osteotomy may be more suitable for patients with a short or retruded chin, since it allows forward migration of the chin and vertical augmentation with bone grafting when necessary.

Both surgical approaches effectively improved facial proportions without altering midface height. The ratio of chin width to chin height was an important indicator for evaluating aesthetic improvement after surgery^7^. Our data suggest that the total mandibular inferior border circumferential osteotomy provides a more refined and symmetrical lower facial contour, especially in patients with facial asymmetry or excessive mandibular width.

These findings are consistent with previous studies reporting that full mandibular inferior border circumferential osteotomy and T-chinoplasty combined with mandibular angle osteotomy are effective in correcting mandibular deformities and enhancing facial appearance^8,9^. By adjusting the mandibular angle and lower border, both techniques can harmonize jaw dimensions, correct asymmetry, and restore facial balance^10^. Moreover, the improvement of mandibular contour contributes to enhanced self-confidence and social well-being in patients^11^.

Although both surgical techniques yielded favorable aesthetic outcomes, potential risks must be considered. In this study, the incidence of postoperative complications—such as lower lip numbness, hematoma, infection, and soft tissue ptosis—did not differ significantly between the two groups. All complications were minor and resolved with conservative management, underscoring the safety and clinical feasibility of both procedures. Nonetheless, thorough preoperative assessment and careful surgical planning are essential to minimize risks^12^. Moreover, the improvement of mandibular contour contributes to enhanced self-confidence and social well-being in patients^13,14^. Future multicenter studies with larger cohorts and standardized surgical protocols are recommended to confirm these findings^15^.

Despite its strengths, this study has certain limitations. The relatively small sample size may limit the generalizability of the results, and longer follow-up periods are required to evaluate the long-term stability of outcomes. Additionally, subtle differences in surgical execution among operators could influence the results. Future multicenter studies with larger cohorts and standardized surgical protocols are recommended to confirm these findings and explore long-term morphological changes.

## CONCLUSION

In conclusion, both total mandibular inferior border circumferential osteotomy and T-chinoplasty combined with mandibular angle osteotomy are effective methods for improving mandibular contour and correcting facial asymmetry. Total mandibular inferior border circumferential osteotomy provides greater chin narrowing and superior alignment of the facial midline, making it more suitable for patients with long, wide, or asymmetric lower faces. Conversely, T-chinoplasty combined with mandibular angle osteotomy remains advantageous for patients requiring anterior advancement or vertical augmentation of the chin. Ultimately, surgical selection should be individualized, emphasizing patient-specific morphology and aesthetic expectations. Close communication between the surgeon and patient is critical for achieving optimal and satisfactory outcomes. Further research with larger sample sizes and extended follow-up is warranted to validate these conclusions.

## Data Availability

The datasets generated and/or analyzed during the current study are available from the corresponding author upon reasonable request.
